# Positive Aspects of Caregiving Among Family Caregivers of Individuals With Dementia

**DOI:** 10.1093/geroni/igaa057.1153

**Published:** 2020-12-16

**Authors:** Mary Grace Asirot, Anna Papazyan, Yeonsu Song

**Affiliations:** 1 UCLA, Los Angeles, California, United States; 2 VA Greater Los Angeles Healthcare System, North Hills, California, United States

## Abstract

Traditionally, caregiving for individuals with dementia has been viewed as a negative experience. Understanding positive aspect of caregiving and related factors is important to improve health among family caregivers. We analyzed baseline data from an ongoing dyadic sleep education trial for individuals with dementia and their caregivers (N=21 dyads; mean age 70.8± 11.1 for caregivers, 80.5± 8.3 for care-recipients). The Positive Aspects of Caregiving (PAC 9-item) was used to assess subjective satisfaction with caregiving. Other measures included Zarit Burden Interview (ZBI), SF-12 Health Survey (SF-12v2), Revised Memory and Behavior Problems Checklist (RMBPC), and Pittsburgh Sleep Quality Index (PSQI). Pearson correlations and t-tests were calculated for analyses. Caregivers most frequently endorsed that caregiving enabled them to appreciate life more (n=16 agreed a lot). Caregivers who began providing care within the first few months of the care-recipient needing care (n=16) had greater positive aspects of caregiving than those who started providing care sometime later (n=5) (36.37±7.33 versus 25.8±8.29, p=0.01). Caregivers with higher PAC scores had lower ZBI score (r=-0.49, p=0.02), better mental health on the SF-12v2 (r=0.53, p=0.01), less distress related to care-recipient behaviors on the RMBPC (r=-0.50, p=0.02), and lower PSQI subscale (perceived sleep quality) score (r= -0.46, p=0.04). Findings suggest that higher positive caregiving experience was associated with better mental health and sleep quality, and less burden and distress from the care-recipients behaviors. More research is needed to better understand this relationship and to determine possible interventions to increase positive aspects of caregiving.

